# Temporal variation in translocated Isle Royale wolf diet

**DOI:** 10.1002/ece3.9873

**Published:** 2023-03-16

**Authors:** Adia R. Sovie, Mark C. Romanski, Elizabeth K. Orning, David G. Marneweck, Rachel Nichols, Seth Moore, Jerrold L. Belant

**Affiliations:** ^1^ Department of Fisheries and Wildlife Michigan State University East Lansing Michigan USA; ^2^ National Park Service Isle Royale National Park Houghton Michigan USA; ^3^ College of Environmental Science and Forestry State University of New York Syracuse New York USA; ^4^ Conservation Alpha Johannesburg South Africa; ^5^ Department of Biology and Environment Grand Portage Band of Lake Superior Chippewa Grand Portage Minnesota USA

**Keywords:** beaver, diet, moose, predation, scat analysis, wolf

## Abstract

Wolves (*Canis lupus*) can exert top‐down pressure and shape ecological communities through the predation of ungulates and beavers (Castor spp.). Therefore, understanding wolf foraging is critical to estimating their ecosystem‐level effects. Specifically, if wolves are consumers that optimize tradeoffs between the cost and benefits of prey acquisition, changes in these factors may lead to prey‐switching or negative‐density dependent selection with potential consequences for community stability. For wolves, factors affecting cost and benefits include prey vulnerability, risk, reward, and availability, which can vary temporally. We described the wolf diet by the frequency of occurrence and percent biomass and characterized the diet using prey remains found in wolf scats on Isle Royale National Park, Michigan, USA, during May–October 2019 and 2020. We used logistic regression to estimate prey consumption over time. We predicted prey with temporal variation in cost (availability and/or vulnerability) such as adult moose (*Alces alces*), calf moose, and beaver (*Castor canadensis*) to vary in wolf diets. We analyzed 206 scats and identified 62% of remains as beaver, 26% as moose, and 12% as other species (birds, smaller mammals, and wolves). Adult moose were more likely to occur in wolf scats in May when moose are in poor condition following winter. The occurrence of moose calves peaked during June–mid‐July following birth but before calf vulnerability declined as they matured. By contrast, beaver occurrence in wolf scat did not change over time, reflecting the importance of low‐handling cost prey items for recently introduced lone or paired wolves. Our results demonstrate that the wolf diet is responsive to temporal changes in prey costs. Temporal fluctuation in diet may influence wolves' ecological role if prey respond to increased predation risk by altering foraging or breeding behavior.

## Graphical abstract

We analyzed wolf diet from scats in relation to optimal foraging. We found 62% of remains as beaver, 26% as moose, and 12% as other (birds, smaller mammals, and wolves). The contents of wolf scat changed over time. Wolf diet is plastic and responsive to temporal changes in prey acquisition cost as predicted by optimal foraging theory.

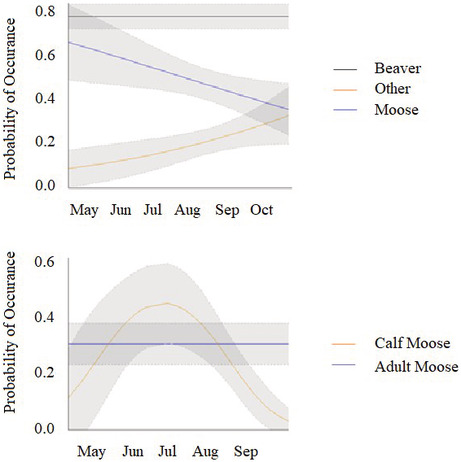

## 1 INTRODUCTION

Predation can shape ecosystems by controlling prey population abundance and influencing where prey forage, resulting in landscape scale changes to vegetation and nutrient flows (Estes et al., 2011; Halpern et al., 2006; Terborgh et al., 2001). Specifically, wolf (*Canis lupus*) predation can produce top‐down structuring in many systems (Bump et al., 2009; Ripple & Beschta, 2012). While predation can influence prey activity (Gaynor et al., 2019; Kohl et al., 2018; Suraci et al., 2016), it is less clear whether prey demography and behavior can drive wolf foraging decisions (Abrams, 1992; Katz et al., 2015). Consumers should optimize caloric intake by balancing the calories gained from a food item and the caloric and safety costs of searching for and handling the item (Pyke et al., 1977). For wolves, the caloric benefits of prey items are relatively stable while the costs associated with acquisition [search time, subjugation difficulty, and risk of injury] change temporally. This may cause wolf foraging patterns to change over a season, resulting in prey switching with the potential for cascading ecological effects (Basille et al., 2013; Garrott et al., 2007; Latham et al., 2013). Understanding how wolves respond to variations in prey abundance and vulnerability is important for predicting and managing their ecological impacts in harvested and protected populations.

For wolves, the cost of prey acquisition includes search and handling time, as well as injury risk (Griffiths, 1980; Mukherjee & Heithaus, 2013), factors that can change over weeks or months. Predators often target abundant prey to reduce the caloric costs associated with search time (Griffiths, 1980). The availability of prey can change temporally as species migrate into or out of a feeding range, emerge from hibernation or torpor, or following a birth pulse (Metz et al., 2012; Petroelje et al., 2014). Wolves do not always preferentially attack the most abundant prey, vulnerability and risk also influence wolf foraging decisions (Hoy et al., 2021; Tallian et al., 2017). Larger animals require greater effort and risk to subdue (Griffiths, 1980; MacNulty et al., 2009). In contrast, smaller animals or prey in poor condition may require less effort to subdue and may be less likely to inflict injuries (Krumm et al., 2010; MacNulty et al., 2009). Prey risk and vulnerability change temporally (Metz et al., 2012). For example, ungulates are often nutritionally deficient after winter (Huggard, 1993; Kautz et al., 2020) and young animals develop the speed and endurance necessary to evade capture as they mature (Severud et al., 2019). These temporal shifts in prey demography and behavior may alter the wolf diet.

Recently reintroduced wolves on Isle Royale National Park (IRNP), Michigan, USA provide a unique opportunity to investigate wolf diet. Wolves on IRNP prey on a limited number of species that predictably vary in caloric value, availability, and vulnerability, allowing us to evaluate hypotheses of short‐term prey switching. During the ice‐free season (April–October) wolves in IRNP historically preyed on moose and beavers but also consumed snowshoe hares (*Lepus americanus*), small mammals, and birds (Thurber & Peterson, 1993). Wolves in IRNP consume 4‐7 kg/wolf of biomass a day (Thurber & Peterson, 1993) and should select large prey to maximize caloric intake (Carbone et al., 1999). Adult moose (about 260 kg of digestible biomass) are a high‐calorie prey item but require considerable effort to locate and subdue (Sand et al., 2012). Adult moose are most vulnerable in May due to winter malnutrition but regain fat reserves after green‐up in June and July (Parker, 2003; Tischler et al., 2019). Moose calves (45 kg) are available after birth in late May and are guarded by their mothers for the first few weeks of their lives (Edwards, 1983; Stephens & Peterson, 1984). Moose calves may be more vulnerable to wolf attack in late June and July, as they spend less time with their mothers but are capable of outrunning wolves by September (Severud et al., 2019). Beavers (12 kg) are a low‐risk and abundant food source on IRNP during the ice‐free season. In May following ice‐out and in September before ponds freeze, beavers are vulnerable to wolf predation when they forage on land to repair lodges or restore food caches (Gable, Windels, et al., 2018).

We estimated the wolf diet from scats in IRNP during the ice‐free period in 2019–2020. We restricted our study to the ice‐free season because ground‐based fieldwork on Isle Royale is infeasible in winter and knowledge of the summer diet of wolves is limited. We hypothesized that the wolf diet would reflect a strategy that tracks temporal shifts in prey acquisition costs. Specifically, we predicted that due to their high caloric value, moose would be the primary component of the wolf diet by frequency of occurrence and biomass in scats. We also predicted the presence of moose in the wolf diet will be highest in May and decline thereafter. Further, we predicted that moose calf occurrence in scats would be nonlinear, peaking in late June after birth and declining in late summer as calves grow and become less vulnerable to wolf predation. We predicted that beaver occurrence in the wolf diet would vary with peaks in May and September. Finally, we predicted that other prey (e.g., snowshoe hares, small mammals, and birds) would increase in occurrence in late summer as moose become more difficult to kill and wolves increase their use of alternative prey (Gable, Windels, et al., 2018).

## 2 METHODS

### 2.1 Study area

Isle Royale National Park is a 540‐km^2^ archipelago in Lake Superior, 22 km from mainland Ontario, Canada. Over 99% of the park is designated wilderness and supports mixed boreal forests characteristic of the transition between temperate and boreal zones (Sanders & Grochowski, 2013). Mammals in IRNP include moose, wolves, red foxes (*Vulpes vulpes*), beavers, river otters (*Lontra canadensis*), snowshoe hares, red squirrels (*Tamiasciurus hudsonicus*), muskrats (*Ondatra zibethicus*), deer mice (*Peromyscus maniculatus*,) and several species of bats (Johnson et al., 1982). Mean daily high temperatures in summer (June–August) are 21°C and mean daily high temperatures in winter (December–February) are 3°C. Isle Royale National Park receives an average of 734 mm in annual precipitation. Isle Royale National Park is officially closed from November 1 to April 15 every year. From mid‐January to mid‐March the NPS authorizes scientific research and personnel to access the island using light aircraft.

Moose colonized IRNP in the early 1900s and wolves arrived in the 1940s (Vucetich & Peterson, 2009). Moose and wolf populations fluctuate in response to each other, and wolf predation moderates the effects of moose herbivory on vegetation (McLaren & Peterson, 1994). The long‐term population average was 22 wolves in two to three packs (Peterson & Vucetich, 2017). Since 1980 a combination of disease, accidents, and inbreeding depression led to a decline in wolf abundance (Vucetich et al., 2012), declining from 50 in 1980 to a single nonreproducing father–daughter full sibling pair in 2016 (Hedrick et al., 2017). During 2018 and 2019 IRNP personnel and partner organizations released 19 wolves to supplement the population (Romanski et al., 2020). Eight of these wolves were a family group from Michipicoten Island, Ontario, and the remaining wolves were unassociated and unrelated individuals (Hervey et al., 2021). All translocated wolves received Vectronics or Telonics radio‐collars with on‐board Global Positioning System (GPS) scheduled to attempt a location every 5 h. We estimated the wolf population during the ice‐free season of 2019 to be at least 15 adults and in April 2020 we estimated the population to be about 14 individuals, excluding pups born later that year (Romanski et al., 2020). In the year following introduction, pack cohesion on IRNP was limited, and in the second year about half the wolves appeared associated with two packs (Romanski et al., 2020).

### 2.2 Scat collection and processing

**FIGURE 1:**
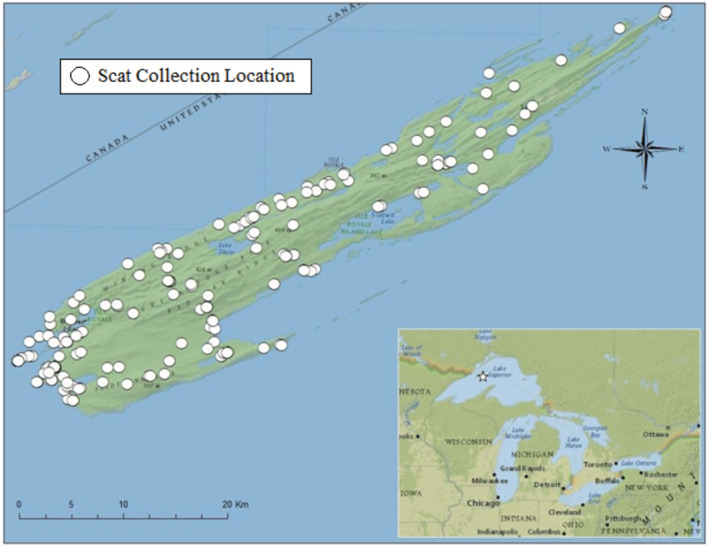
Locations of wolf scats collected (white circles) May–October 2019 and June–September 2020 to determine diet composition, Isle Royale National Park, Michigan, USA.

During 5 May–5 October 2019 and 13 June–22 September 2020 we collected all wolf scats encountered during fieldwork in IRNP. We collected fresh scats (e.g., strong smell, moist, tracks present) at wolf radio‐collar GPS location clusters (Svoboda et al., 2013) and on 265 km of established hiking trails (Figure 1). We placed each scat in a plastic bag, recorded the date and location (NAD83, UTM Zone 16 N), and froze them for later processing. We considered the collection date as the date of deposit, although scats may have been up to 11 days old (Sanchez et al., 2004).

We processed and identified scat contents following Chenaux‐Ibrahim (2015). We placed frozen scats into nylon stockings, washed them in a washing machine to remove digestible material, and then dried the contents in an oven. We spread the contents on a 21.5 × 28 cm plate, overlaid a 25‐point grid, and randomly selected one hair from each point for microscopic identification (Ciucci et al., 2004). A single technician examined all hairs using a compound and dissecting microscope to compare color, shape, diameter, length, and the medulla (the innermost part of the hair). The hairs of ungulates and snowshoe hares have distinct medulla patterns, which allowed for the initial classification of the hair as ungulate, snowshoe hair, or other. We also examined hair scale patterns, especially to differentiate between age classes. We identified hairs using the Adorjan and Kolenosky (1969) reference manual of mammal hair and a reference collection. Before performing hair identification in scats, the technician took a blind test using 100 known hair samples with each expected prey species present, including moose calves (Ciucci et al., 1996), and had 95% identification accuracy.

We could determine moose age class (adult or calf) until 15 September, when the first molt typically occurs (Müller, 2006). After 15 September we identified all moose hair as adult. Wolf hair may be ingested due to grooming or cannibalistic activity, the latter resulting in a higher amount of hair in scats (James, 1983; Müller, 2006). Therefore, we did not consider wolves as prey unless we detected wolf hair at >10% of points in a sample. We categorized all other prey (birds, muskrats, snowshoe hares, small mammals [deer mice and red squirrels], wolves, unidentifiable remains, and vegetation) into an “other” category.

The contents of scats deposited by the same individual or pack may be autocorrelated (Gable et al., 2017; Marucco et al., 2008). To test for spatial autocorrelation, we fit a spline correlogram using the function spline.correlog() in package ncf (Bjornstad & Bjornstad, 2016) of program R (R Core Team, 2022) and considered values <0.2 lacking autocorrelation (Bjornstad & Falck, 2001; McMurry & Politis, 2010). A spline correlogram estimates spatial dependence as a continuous function of distance. We found no indication of spatial autocorrelation in scat contents (SUP 2), and therefore considered all scats as independent samples.

### 2.3 Prey occurrence and biomass

For each scat, we calculated the frequency of occurrence (FO) and percent biomass of each prey item (Mech, 1966; Thurber & Peterson, 1993). We calculated FO as the total number of occurrences on the point grid divided by the total number of points in the sample. As FO can overrepresent small prey and younger animals, we also calculated the percent biomass ingested (Ciucci et al., 1996; Floyd et al., 1978; Klare et al., 2011). To calculate biomass for each species, we multiplied the number of occurrences on the point frame by the correction factor *y* = 0.439 + 0.008 × *x*, where *x* is the digestible mass of the prey item (Floyd et al., 1978). We used the estimated digestible mass of adult moose, calf moose, and beavers on IRNP (Thurber & Peterson, 1993). Due to identification uncertainty, we did not calculate the biomass contribution of species categorized as “other.”

We used logistic regression to estimate temporal variation in the wolf diet. For each scat, we recorded the presence/absence of each prey species and built models representing our hypothesis of how wolf diet may change over time. For each species, we built three models with species presence/absence as the response and constant, linear, or quadratic time as predictors. We considered coefficients from models informative when the 95% confidence interval around the beta estimate did not overlap 0. We used Akaike Information Criterion corrected for small samples (AICc) and AICc weight to select the most parsimonious model (Burnham & Anderson, 2002). We considered models within two AICc units of the best model as competing models unless they were more complex than the top model and the coefficients of the additional variables were uninformative (Burnham & Anderson, 2002).

Due to difficulty identifying moose to age class after 15 September, we ran two sets of models for each statistical approach. The first included all scats with all moose pooled and the second set included scats collected from 5 May to 15 September with moose separated by age class.

## 3 RESULTS

**FIGURE 2:**
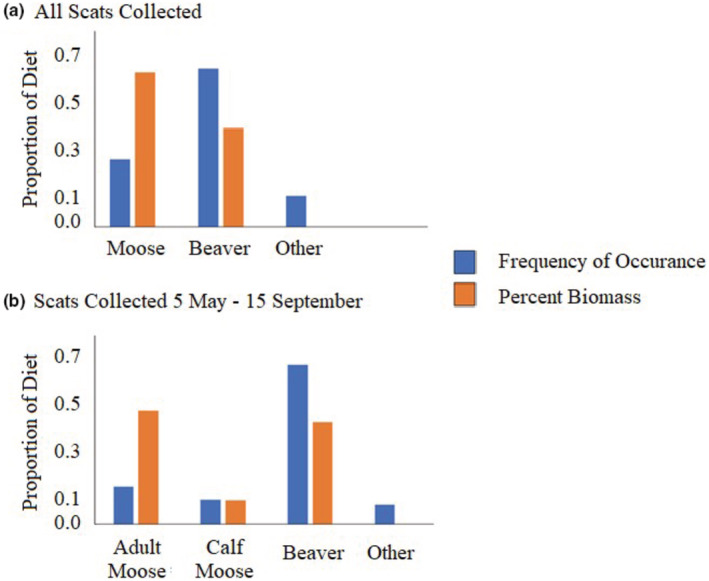
Proportional composition of prey identified in wolf scats, Isle Royale National Park, Michigan, USA by the percent frequency of occurrence (blue bar), and percent biomass (orange bar). (a) Scats collected with moose adults and calves pooled. (b) Scats collected with adult moose and calves separated (15 May −15 September).

We collected 206 scats, 116 in 2019 and 90 in 2020, and detected seven prey items (Table 1). We collected 106 scats at 74 clusters, 43 clusters were created by unaffiliated wolves and 31 were from wolves associating with groups. We collected on average 1.4 scats per cluster (range = 1–7). Scats on average contained 1.6 ± 0.8 standard deviation [SD] items per scat with 56% containing 1 item. We did not detect a difference in prey occurrence in scats between years (*β*_Mooseyear_ = −0.15, se = 0.28; *β*_Beaveryear_ = −0.09, se = 0.34; *β*_Otheryear_ = 0.26, se = 0.34) so we pooled all samples for further analysis. The most frequent prey overall in wolf scats was beaver (62%), followed by moose (26%), and other prey (11%; Figure 2). When we pooled moose age classes, moose comprised 61% of biomass ingested and beaver 39% (Figure 2a). In scats collected from 5 May to 15 September, beaver remained the most common prey item (66%), followed by adult moose (15%), calf moose (10%), and other prey (8%) (Figure 2b). From this subset of scats, beaver and adult moose were the most important prey items by biomass (47% and 42%, respectively), while moose calves represented 10% of the wolf diet. Wolf hairs were uncommon in samples, with one scat comprised entirely of wolf hair (100% of points in the frame).

**TABLE 1:** Occurrence of prey items (hair) identified from 206 wolf (*Canis lupus*) scats collected 5 May–22 October 2019 and 13 June–22 September 2020, Isle Royale National Park, Michigan, USA.

Prey item	Number of points	Number of scats
Beaver	3119	161
Moose		
Moose calf^a^	446	42
Moose adult^a^	697	55
Moose^b^	187	15
Other		
Bird	93	8
Snowshoe hare	118	9
Unknown	444	10
Wolf	85	10
Total	5042	206

^a^ Age class identified for scats collected between 5 May and 15 September.

^b^ Hair identified from scats collected after 15 September or when age could not be identified.

**FIGURE 3:**
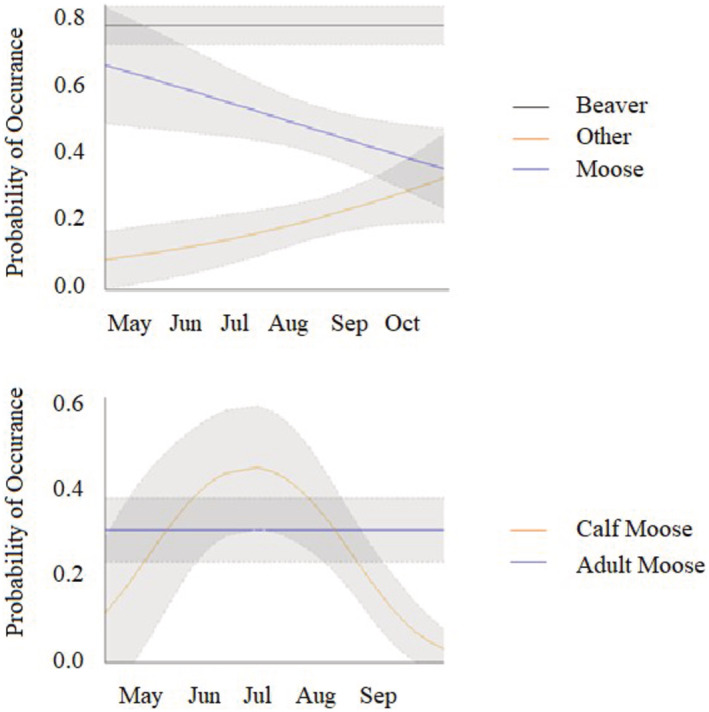
Temporal change in prey occurrence identified from wolf scats, Isle Royale National Park, Michigan, USA. Scats collected with moose adults and calves pooled (top panel) and scats collected with adult moose and calves separated (bottom panel).

Using all scats combined, the linear time model was the most parsimonious for moose and other prey (Table 2). While nonlinear time models were supported, the additional parameters were not informative. The probability of moose hair occurrence declined (*β*_Time_ = −0.31, se = 0.03) from 0.66 in May to 0.36 in October (Figure 3a). The probability of other prey items occurring in scat increased (*β*_Time_ = 0.41, se = 0.19) from 0.09 in May to 0.33 in October (Figure 3a). The most parsimonious model for beaver was constant over time, with the overall probability of occurrence in wolf scats at 0.78 (95% CI = 0.72–0.84) (Figure 3a). The linear time model was also supported, but the additional parameter did not improve parsimony and was not informative.

**TABLE 2:** Logistic regression model selection results for moose (adults and calves pooled), beaver, and other prey occurrences in wolf scats collected 5 May–22 October 2019 and 13 June–22 September 2020, Isle Royale National Park, Michigan, USA.

	Model	K^a^	AICc^a^	ΔAICc^a^	*ω*_*i*_ ^a^	LL^a^
Moose	Linear	2	281.29	0	0.69	−138.62
	Nonlinear	3	283.13	1.84	0.27	−138.5
	Constant	1	287.11	5.82	0.04	−142.55
Beaver	Constant	1	217.30	0	0.44	−107.64
	Linear	2	217.83	0.53	0.34	−106.89
	Nonlinear	3	218.76	1.46	0.21	−106.32
Other	Linear	2	211.74	0	0.47	−103.84
	Nonlinear	3	211.79	0.05	0.46	−102.84
	Constant	1	215.71	3.97	0.06	−106.85

Abbreviations: AICc, Akaike Information Criterion corrected for small sample size; ΔAICc, difference between the AICc value of each model and the lowest AICc model; LL, negative log likelihood; *ω*_*i*_, AICc weight.

^a^ K, number of parameters in model.

When considering moose by age class during 5 May–15 September, the most parsimonious model for adult moose was constant over time. The probability of adult moose hair in scats was 0.30 (95% CI = 0.23–0.37) (Table 3, Figure 3b). By contrast, the best model for the presence of calf hair in scats was nonlinear, with the probability of calf hair greatest in the first week of July (0.44, 95% CI = 0.30–0.58) and declining through mid‐September (*β*_Time_ = 7.11, se = 2.58; *β*_Time_^2^ = −7.75, se = 2.65).

**TABLE 3:** Logistic regression model selection results for adult and calf in wolf scats collected 5 May–15 September 2019 and 13 June–15 September 2020, Isle Royale National Park, Michigan, USA.

	Model	K^a^	AICc^a^	ΔAICc^a^	*ω*_*i*_ ^a^	LL^a^
Adult	Constant	1	192.95	0	0.66	−95.46
	Linear	2	195	2.05	0.24	−95.46
	Nonlinear	3	196.73	3.78	0.1	−95.29
Calf	Nonlinear	3	163.28	0	0.98	−78.56
	Linear	2	171.83	8.55	0.01	−83.87
	Constant	1	175.24	11.97	0	−86.61

Abbreviations: AICc, Akaike Information Criterion corrected for small sample size; ΔAICc, difference between the AICc value of each model and the lowest AICc model; LL, negative log likelihood; *ω*_*i*_, AICc weight.

a K = number of parameters in model.

## 4 DISCUSSION

We found that wolf foraging decisions in IRNP appeared influenced by the availability and vulnerability of their prey. As we predicted, wolves on Isle Royale shifted their diet in response to prey vulnerability and availability. Viewing the wolf diet in relation to variable acquisition costs could explain why the diet of wolves in IRNP diverged from previous studies. In North America and Europe, large‐ and medium‐size ungulates comprise >60% of the wolf diet by the frequency of occurrence (Carbone et al., 1999; Derbridge et al., 2012; Newsome et al., 2016; Theuerkauf, 2009). By contrast, in IRNP ungulates comprised only 26% of the wolf diet by the frequency of occurrence. Our findings likely reflect the relative availability and vulnerability of moose and beaver in IRNP. Beavers in IRNP are at historically high densities of >1 colony/km^2^ (NPS unpublished data; Smith & Peterson, 2021) and wolves can ambush and subdue beavers in less than 5 min (Gable, Stanger, et al., 2018). By contrast, moose density (~3.7/km^2^) in IRNP is within its historic [1960–2020] range (Smith & Peterson, 2021). Also, wolves often exert considerable energy to subdue moose, chasing them up to 1 km (Mech et al., 1995; Paquet, 1989). While moose are an order of magnitude more calorically profitable than beavers, the abundance and ease of capturing beavers make them an important prey of wolves in IRNP. Wolves in IRNP appear to select prey (i.e., beavers) that are highly available (benefit) and easier to catch (lower costs) than larger ungulates (i.e., moose).

Contrary to our prediction that beaver would be an important secondary and temporally variable food item, wolves on Isle Royale consumed beaver at high rates throughout the ice‐free season. The high amount of beaver consumption we observed is atypical for IRNP (Thurber & Peterson, 1993) and wolves in general (Newsome et al., 2016) and likely reflects high beaver availability and vulnerability in conjunction with weak wolf pack formation. Beaver densities are at historic high levels with 1 colony/km^2^ (Smith & Peterson, 2021) compared with a mean of 0.28 colony/km^2^ from 1962 to 2008 when beavers comprised only 14% of biomass of the wolf diet in IRNP (Gable, Windels, Romanski et al., 2018; Romanski, 2010). High beaver densities likely reduce wolf search time and may increase beaver vulnerability as they forage farther from water as palatable trees near ponds become limited (Gable, Stanger, et al., 2018; Gable & Windels, 2018; Gable, Windels, et al., 2018). In the year following introduction, pack cohesion on IRNP was limited, and in the second year about half the wolves appeared associated with a pack (Romanski et al., 2020). Historically, the percentage of solitary wolves on IRNP rarely exceeded 20% of the population (Thurber & Peterson, 1993); smaller prey may be less risky for individual or small groups of wolves to attack (Escobedo et al., 2015). Pack cohesion increased during 2021–2023 (NPS, unpublished data), and pack stabilization may result in a dietary shift in this population.

While beavers were the most frequently consumed prey item, moose contributed 50% more biomass to the wolf diet. Moose comprised most of the biomass ingested by wolves, which supported our predictions; however, moose comprised less of the wolf diet than we expected. In the Great Lakes Region of North America, ungulates comprise >80% of the wolf diet and historically (1975–1989) 85%–95% of biomass consumed by IRNP wolves (Newsome et al., 2016; Thurber & Peterson, 1993). This shift in wolf diet may be a result of high beaver density, moose population size and age structure, limited pack formation, or a combination of these and other factors. Prey age structure can impact wolf kill rates as moose calves and adults >10 years old are most vulnerable to wolf predation (Montgomery et al., 2013; Sand et al., 2012). Our temporal analyses highlight the importance of available vulnerable moose (i.e., nutritionally deficient adults in early spring and calves after birth) and a lack of vulnerable moose may skew the wolf diet on IRNP. Further, the likely limited pack cohesion in recently introduced wolves (NPS, unpublished data) may account for the low occurrence of moose in the diet. Cooperative hunting increases the efficiency of capturing larger prey, and there is a positive correlation between group size and prey size among social carnivores (Macdonald, 1983; MacNulty et al., 2014). While paired and single wolves can kill moose (Thurber & Peterson, 1993), increased pack size can improve the wolf success rate for difficult to capture prey (MacNulty et al., 2014). For paired or single wolves, hunting beaver in IRNP is less risky and potentially as energetically efficient as hunting moose. As wolves establish packs and form more cohesive social bonds, their diet may change to reflect historic trends.

In accordance with our predictions, adult moose were more likely to occur in wolf scat early in the ice‐free season, consistent with previous studies demonstrating the importance of individuals in poor condition in the wolf diet (Hoy et al., 2021; Stahler et al., 2006). In addition, the prevalence of moose in the wolf diet early in the ice‐free season may indicate the importance of scavenging starving or winter tick (*Dermacentor albipictus*)‐infested individuals (Forbes & Theberge, 1992). Our results suggest that scavenging may be a common early summer strategy in wolf‐moose systems (Forbes & Theberge, 1992; Huggard, 1993; Messier & Crête, 1985). These early‐season moose may also represent partially consumed and cached winter kills (Vucetich et al., 2012).

The occurrence of moose calf hair in wolf scats peaked in late June as we predicted. However, moose calves made up less of the wolf diet than we expected with calves comprising 10% of the biomass ingested, similar to rates reported by Thurber and Peterson (1993). This amount of consumption of calves is low for wolves, which tend to select for juvenile ungulates (Husseman et al., 2003; Mattioli et al., 2011) and calves can comprise 60%–90% of biomass ingested in some moose‐wolf systems (Sand et al., 2008; Wam & Hjeljord, 2003). Potential greater cow vigilance in IRNP (Edwards, 1983; Stephens & Peterson, 1984) could result in a shorter period of calf vulnerability than in other systems, reducing the consumption of calves by wolves. Interestingly, our results differed from Hoy et al. (2021) who reported strong, negative frequency‐dependent selection for moose calves by IRNP wolves in winter. This divergence in seasonal diets may be a result of the larger prey base available to wolves in the ice‐free season. The availability and vulnerability of beaver also may alleviate predation pressure on moose calves.

As moose became more difficult to capture, the occurrence of less calorically valuable prey (snowshoe hares, small mammals, and birds, categorized as “other”) increased in wolf scats. Our results highlight the importance of alternative prey in supporting wolves through resource‐limited periods. Prey switching can allow higher densities of wolves to persist on a landscape and alter the population and behavior of tertiary prey (Garrott et al., 2007; Latham et al., 2013). Snowshoe hares are an important herbivore on Isle Royale (Belovsky, 1984) and increased wolf predation of hares in late summer could help limit the effects of hare browsing on vegetation.

Our results may be limited by the inherent difficulty of estimating predator diets in forested landscapes. Scat analysis provides advantages over tracking or GPS cluster investigation to document small or rare items in carnivore diets (Klare et al., 2011). However, scat analysis is prone to observer error, can overrepresent small prey, assumes constant scat deposition rates, and biomass calculations rely on strong assumptions of carcass use (Klare et al., 2011; Massey et al., 2021; Spaulding et al., 2000). Specifically, in IRNP wolves often scavenge and partially consume carcasses (Vucetich et al., 2012), which could bias our biomass calculations. Also, due to the difficulty of identifying moose calves after the first molt, we assumed all moose hair in scats after 15 September were from adults. This assumption may inflate the biomass of moose consumed after 15 September as these could include young of the year post molt. Despite this potential inflation of our biomass estimate, we still found that moose comprised less of the wolf diet than in previous studies, likely reflecting a decreased use of moose in late summer. Finally, because of our opportunistic scat collection, some individuals may be overrepresented (i.e., radio‐collared wolves and their pack members). However, our study was unique in that all but two adult wolves in IRNP were radio‐collared at the time of our study and contributed to GPS clusters, increasing the probability that we sampled the entire population.

Wolves appear to optimize tradeoffs between the costs and benefits of prey acquisition temporally, dynamically responding to changes over time. Just as prey respond to seasonal variability in predation risk, we found wolves responded to seasonal changes in prey availability and vulnerability (Basille et al., 2013; Garrott et al., 2007; Latham et al., 2013). The tendency of wolves to prey switch in response to changes in prey availability is unclear, with some studies indicating negative frequency dependence (Hoy et al., 2021; Tallian et al., 2017) and other studies suggesting prey‐switching (Garrott et al., 2007; Latham et al., 2013). We found that wolves appeared to shift their diet in response to prey availability and vulnerability, supporting the prey‐switching hypothesis. The dynamic summer foraging behavior of wolves may have important cascading and landscape consequences. Specifically, the high rate of beaver predation may influence landscape‐level change in IRNP. Beavers are at historically high densities in IRNP, and the wetlands they create can dramatically alter landscape‐level water and nutrient flow (Johnston, 2014; Rosell et al., 2005). Wolf predation could reduce the number and duration of these impoundments (Gable et al., 2020) and restore interrupted ecological functions in these areas. Further, the relatively low occurrence of moose in wolf diets suggests that when vulnerable alternate prey are available, wolves may not exert top‐down regulation on moose populations.

Our results suggest that limited pack size, increased frequency of lone wolves, and disrupted pack cohesion can result in wolves selecting for smaller (i.e., less risky) and more abundant prey. Understanding these dynamics could have implications for wolf populations, or other large, social carnivores, under varying management scenarios (e.g., reintroductions, harvested populations, genetic augmentation, depredation management) that could potentially disrupt social structure (Haber, 1996; Rutledge et al., 2010) similar to the recently reintroduced wolves of IRNP. Ecological restoration involving large carnivores and their primary prey could be improved by incorporating the abundance and vulnerability of alternative prey into project planning and implementation.

## AUTHOR CONTRIBUTIONS

**Adia Sovie:** Formal analysis (lead); writing – original draft (equal); writing – review and editing (equal). **Mark Romanski:** Conceptualization (equal); funding acquisition (lead); writing – original draft (equal); writing – review and editing (equal). **Elizabeth Orning:** Data curation (lead); investigation (lead); methodology (lead); writing – review and editing (equal). **David Mareweck:** Investigation (equal); writing – review and editing (equal). **Rachel Nichols:** Investigation (supporting); writing – review and editing (supporting). **Seth Moore:** Resources (lead). **Jerrold Belant:** Conceptualization (equal); project administration (equal); supervision (equal); writing – original draft (equal); writing – review and editing (equal).

## Supporting information

Sup 1. Distribution of scat samples collected by month 5 May‐15 September 2019 and 13 June‐15 September 2020, IsleRoyale National Park, Michigan, USA.
Sup 2. Estimated spatial correlation function with 95% confidence intervals for moose (A) beaver (B), and other prey (C), 5 May‐15 September 2019 and 13 June‐15 September 2020, Isle Royale National Park, Michigan, USA.

## Data Availability

Data are available on Dryad doi:10.5061/dryad.djh9w0w3k.
