# Supplementary material for: Temporal variation in translocated Isle Royale wolf diet
Source: Ecol Evol. 2023 Mar 16;13(3):e9873. doi: 10.1002/ece3.9873 (PMC10019911; doi:10.1002/ece3.9873)
Supplement: Supplementary file 1 — Sup 1. Distribution of scat samples collected by month 5 May‐15 September 2019 and 13 June‐15 September 2020, IsleRoyale National Park, Michigan, USA. Sup 2. Estimated spatial correlation function with 95% confidence intervals for moose (A) beaver (B), and other prey (C), 5 May‐15 September 2019 and 13 June‐15 September 2020, Isle Royale National Park, Michigan, USA. [file ECE3-13-e9873-s001.docx]

Sup 1. Distribution of scat samples collected by month 5 May–15 September 2019 and 13 June–15 September 2020, Isle Royale National Park, Michigan, USA.


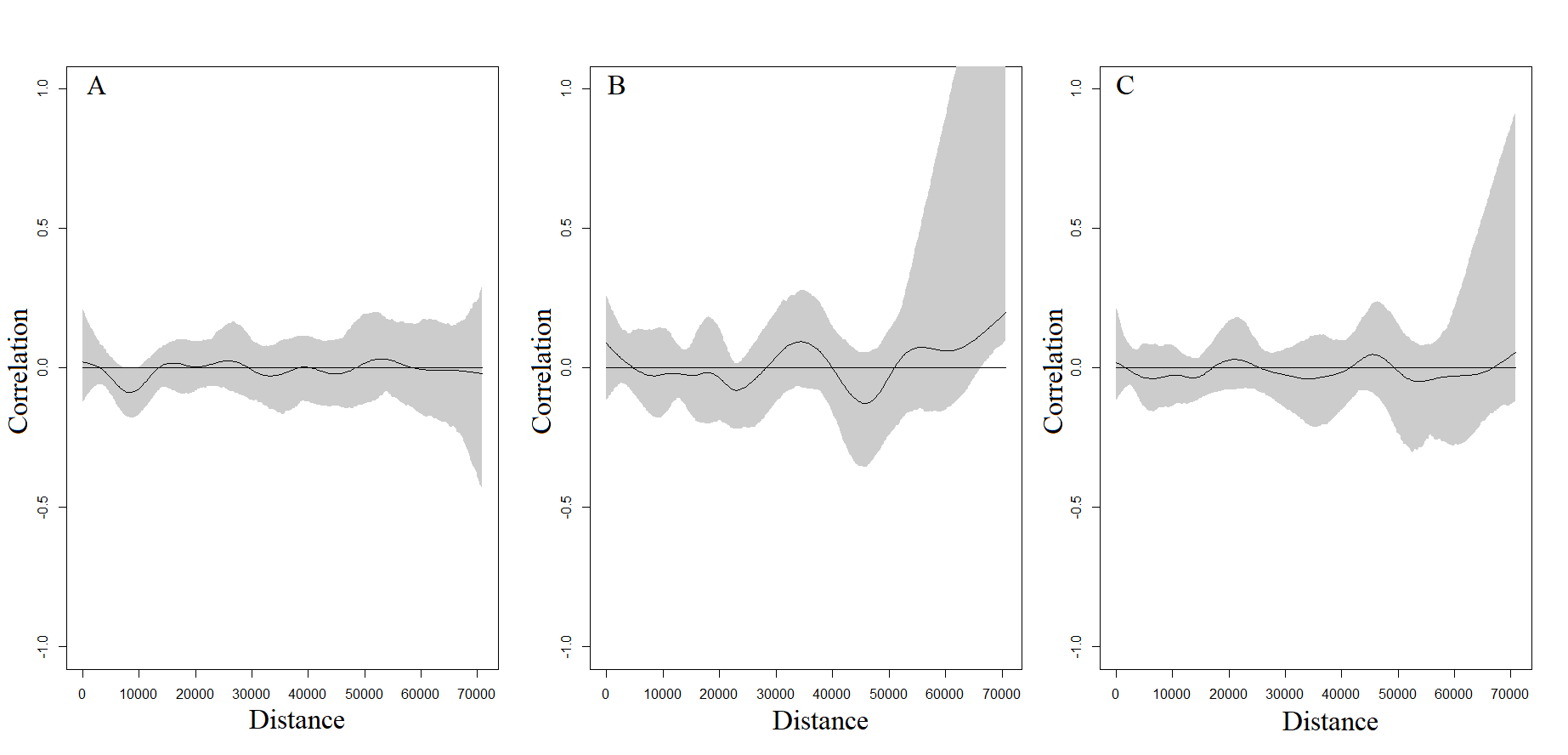


Sup 2. Estimated spatial correlation function with 95% confidence intervals for moose (A) beaver (B), and other prey (C), 5 May–15 September 2019 and 13 June–15 September 2020, Isle Royale National Park, Michigan, USA.
